# Roles of immunoglobulin GM and KM allotypes and Fcγ receptor 2 A genotypes in humoral immunity to a conserved microbial polysaccharide in pulmonary diseases

**DOI:** 10.1038/s41435-024-00318-y

**Published:** 2025-01-07

**Authors:** Janardan P. Pandey, Paul J. Nietert, Aryan M. Namboodiri, Christine Kimball, Patrick A. Flume

**Affiliations:** 1 Department of Pharmacology & Immunology, Medical University of South Carolina, Charleston, SC USA; 2 Department of Public Health Sciences, Medical University of South Carolina, Charleston, SC USA; 3 Department of Medicine, Medical University of South Carolina, Charleston, SC USA

**Keywords:** Immunology, Genetic association study

## Abstract

Immunoglobulin GM (γ marker) and KM (κ marker) allotypes—encoded by immunoglobulin heavy chain G (*IGHG*) and immunoglobulin κ constant (*IGKC*) genes—have been shown to be associated with immune responsiveness to a variety of self and nonself antigens. The aim of the present investigation was to determine whether allelic variation at the GM and KM loci was associated with antibody responsiveness to poly-N-acetyl-D-glucosamine (PNAG), a broadly-conserved surface polysaccharide expressed by many microbial pathogens. In addition, we wished to determine whether Fcγ receptor 2 A (*FCGR2A*) genotypes, which have been shown to be risk factors for some pathogens, also influenced antibody responses to PNAG. DNA from 257 patients with various pulmonary diseases (PD) was genotyped for several GM, KM, and *FCGR2A* alleles, and plasma were characterized for anti-PNAG IgG antibodies. The levels of IgG4 antibodies to PNAG were associated with *FCGR2A* genotypes (*p* = 0.01). Also, KM and *FCGR2A* alleles epistatically contributed to anti-PNAG IgG3 antibody responses: subjects with KM 1/1 or KM 1/3 and homozygous for the R allele of *FCGR2A* had the highest levels of anti-PNAG IgG3 antibodies compared to all other genotype combinations. If confirmed by larger studies, these results are potentially relevant to immunotherapy against many PNAG-expressing infectious pathogens.

## Introduction

Emergence of novel pathogens and the evolution of antibiotic-resistant bacteria have underscored the importance of immunotherapeutic approaches for the treatment of bacterial diseases. Vaccination, a major contributor to human health in the twentieth century, has been successful against many bacterial pathogens. However, for many pathogens relevant to pulmonary diseases (PD), such as non-tuberculous mycobacteria (NTM)—whose prevalence in patients with cystic fibrosis (CF) is 10,000-fold higher than in the general population—no vaccines are currently available [1].

Selection of an appropriate immunogen, which can induce broadly neutralizing antibodies endowed with vigorous immune effector functions, is one of the most important challenges in the development of a vaccine. It would be preferable if the antigen/immunogen were broadly conserved across multiple pathogens, thus potentially generating immunity against many infectious agents. Poly-N-acetyl-D-glucosamine (PNAG) appears to meet these criteria. It is a broadly-conserved surface polysaccharide expressed by many microbial pathogens, including NTM *Mycobacterium avium* and *M. abscessus*, *Streptococcus pneumoniae*, *Staphylococcus aureus, Aspergillus fumigatus*, and many others [2]. Naturally-occurring anti-PNAG antibodies are present in healthy individuals, but they are not protective against infection [3]. Identification and understanding of the host factors that influence naturally occurring immune responses to PNAG are important prerequisites to successfully designing a vaccine or therapeutic antibodies against pathogens expressing this polysaccharide antigen.

There is tremendous interindividual variability in the outcome of any infection, suggesting the involvement of host genetic factors. Immunoglobulin GM (γ marker) and KM (κ marker) allotypes—encoded by immunoglobulin heavy chain G (*IGHG*) and immunoglobulin κ constant (*IGKC*) genes on chromosomes 14 and 2, respectively—have been shown to be associated with susceptibility to several infectious, autoimmune, and malignant diseases and with immune responsiveness to a variety of self and nonself antigens [4–6]. Of specific relevance here, GM 23 allotype has been shown to be associated with IgG2 subclass-specific responses to a polysaccharide vaccine [7] and KM 1,3 allotypes with whole IgG response to PNAG in CF patients [8].

The aim of the present investigation was to determine whether allelic variation at the GM and KM loci is associated with antibody responsiveness to PNAG in PD patients. In addition, we wished to determine whether Fcγ receptor 2 A (*FCGR2Α*) genotypes, which have been shown to be risk factors for some CF pathogens [9], also influenced antibody responses to PNAG in PD patients. It is relevant to note that allelically disparate FcγR 2 A receptors are known to bind IgG of all subclasses with differing affinities [10].

## Materials and methods

### Specimens

DNA and plasma samples were obtained from the NTM Biorepository of the Medical University of South Carolina. The samples were derived from 257 PD patients: CF = 46, non-CF bronchiectasis = 150, other lung diseases = 61. This study cohort is enriched for subjects known to have NTM-PD, but other pathogens expressing PNAG are also involved.

### Genotyping

IgG1 markers GM 3 and 17 (arginine to lysine) and IgG2 markers GM 23− and 23+ (valine to methionine), were determined by a previously described TaqMan® genotyping assay [8]. The κ chain determinants KM 1 and 3 were characterized by a previously described polymerase chain reaction-restriction fragment length polymorphism (PCR-RFLP) technique [11]. Genotyping of FCGR2Α histidine (H)/arginine (R) alleles was performed by RT-PCR, using pre-designed TaqMan® genotyping assays from Applied Biosystems Inc.

### Measurement of anti-PNAG antibodies

Antibodies to PNAG were measured by a previously described ELISA, using plasma from patients in the NTM biorepository [8]. The results were expressed as absorbance units (AU)/μL.

### Statistical analyses

Demographic characteristics and the distribution of genotypes across the 3 groups of PD patients (CF, non-CF bronchiectasis, other lung diseases) were reported and compared using chi-square tests, Fisher’s exact tests, or Kruskal-Wallis tests, as appropriate. Race was defined according to the medical record entry, and categorized as ‘White’, ‘Black’, or ‘Other’. Anti-PNAG IgG antibody levels were compared across disease groups using non-parametric Kruskal-Wallis tests, due to their skewed (non-normal) distributions. To examine the additive effect of each minor allele of interest on IgG antibody levels, quantile regression models (QRMs) were constructed. Quantile regression was selected based on the lack of normality in the IgG antibody levels, the ability of the models to incorporate additive allelic effects, and the ability to include disease group as a covariate. QRMs were also constructed to examine potential pairwise interactions among the four genes of interest, while adjusting for disease group. For these interaction models, genotypes were dichotomized by combining the least frequently occurring homozygous group with the heterozygous group. Dichotomization was used to reduce model instability due to small numbers of subjects with two copies of the minor allele for both genes involved in the potential pairwise interaction. For all analyses, *p* < 0.05 was considered statistically significant, and no adjustment was made for multiple testing, owing to the fact that this was an exploratory/hypothesis generating study [12]. Analyses were conducted using SAS v9.4 (SAS Institute, Cary, NC).

## Results

Table 1 presents the descriptive statistics for the study population demographics and genotype distributions, stratified by disease type. Patients with CF were significantly (*p* < 0.05) younger (mean = 35.5 ± 14.0 years) and more likely to be male (52.2%) than patients with non-CF bronchiectasis (67.0 ± 10.8 years, 12.7% male) and patients with other lung diseases (63.1 ± 13.7 years, 24.6% male). The large majority (93%) of all patients were white; black patients comprised 3.9% of patients, and 6.6% of patients were reported as belonging to other race groups. There were no significant differences in the distribution of the four genes of interest among the three disease groups.

**Table 1:** Descriptive statistics for the study population, stratified by disease type.

Variable	Statistic	Subjects with CF (*n* = 46)	Subjects with non-CF bronchiectasis (*n* = 150)	Subjects with other lung disease (*n* = 61)
Age (y)^a^	Mean (SD)	35.5 (14.0)	67.0 (10.8)	63.1 (13.7)
Sex^a^	% male	52.2%	12.7%	24.6%
Race				
White	%	97.8%	92.7%	91.8%
Black	%	0.0%	2.7%	4.9%
Other	%	2.2%	4.7%	3.3.%
GM 3/17 genotypes				
3/3	%	41.3%	47.3%	48.3%
3/17	%	45.7%	41.2%	43.3%
17/17	%	13.0%	11.5%	8.3%
GM 23 genotypes				
+/+	%	17.4%	21.1%	20.0%
+/−	%	45.7%	45.6%	45.0%
−/−	%	37.0%	33.3%	35.0%
FCGR2A genotypes				
H/H	%	10.9%	27.0%	26.7%
H/R	%	54.4%	44.6%	55.0%
R/R	%	34.8%	28.4%	18.3%
KM genotype				
1/1	%	4.4%	1.4%	3.3%
1/3	%	17.4%	12.9%	14.8%
3/3	%	78.3%	85.7%	82.0%

^a^Indicates a significant (*p* < 0.05) difference among groups chi-square, Fisher’s exact, or Kruskal-Wallis test, as appropriate.

**Fig. 1:**
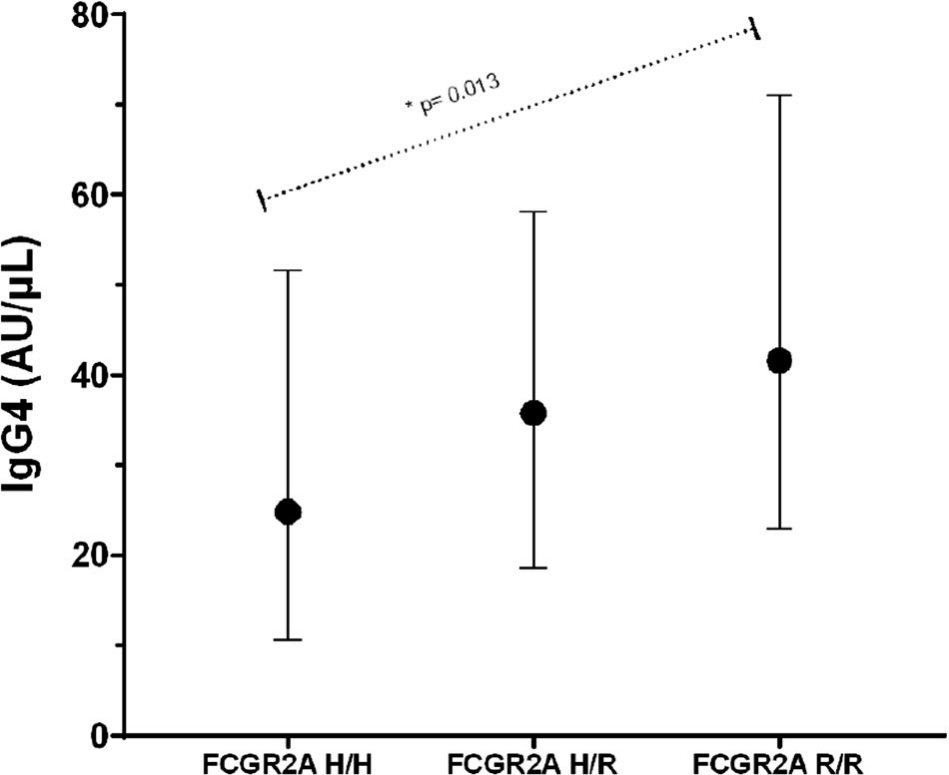
Association between FCGR2A and IgG4 Levels. IgG4 levels are summarized for each FCGR2A genotype (H/H, H/R, R/R). Circles represent medians, and the error bars extend from the 25th to the 75th percentiles. Results of the quantile regression model suggest a statistically significant (*p* = 0.013) additive effect of the minor allele (R) on IgG4 levels, meaning in this case that more copies of the R allele are associated with higher IgG4 levels.

**Fig. 2:**
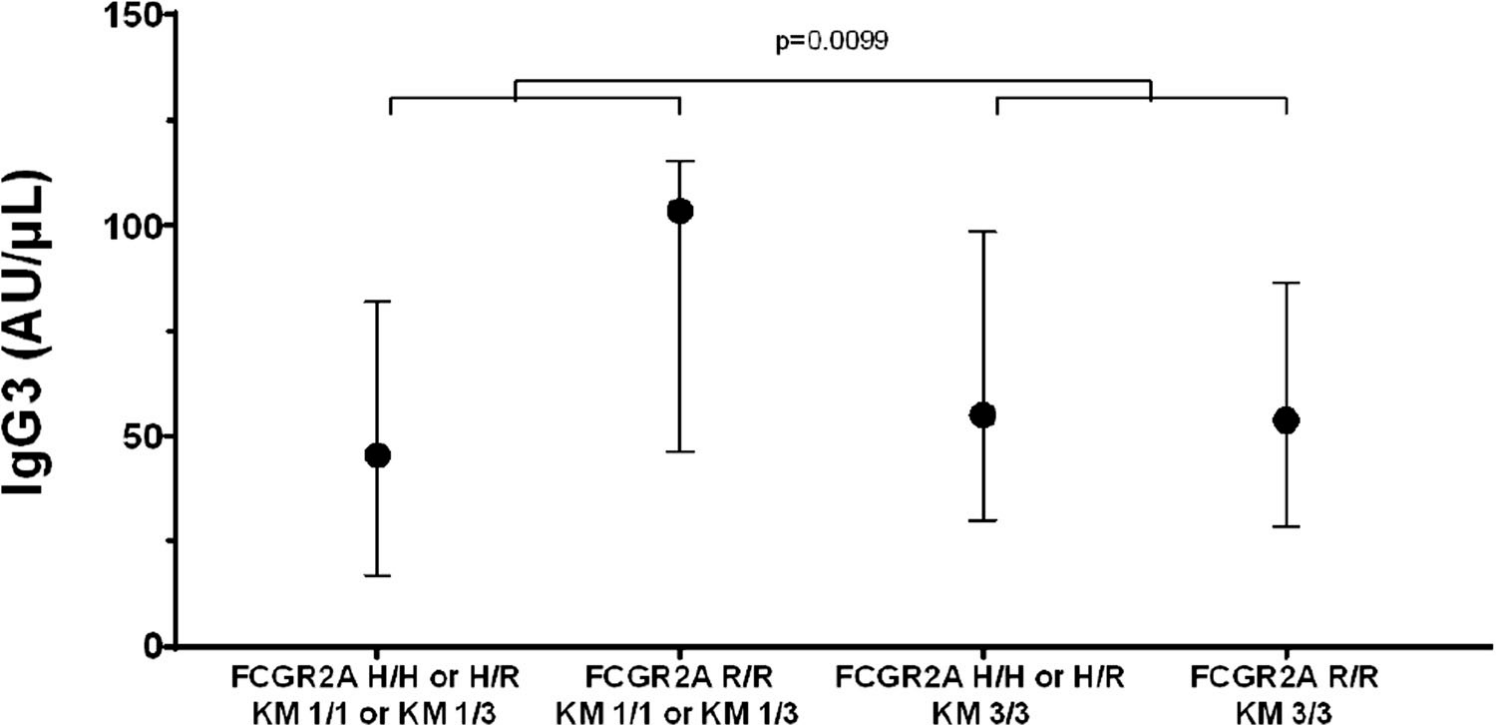
Potential interaction between FCGR2A and KM genotypes on IgG3 Levels. IgG3 levels are summarized for each combination of binarized FCGR2A and KM genotypes. Circles represent medians, and the error bars extend from the 25th to the 75th percentiles. Results of the quantile regression model suggest a statistically significant (*p* = 0.0099) interaction between these genes on IgG3 levels. In this case, there was a marked difference in the IgG3 medians among patients with and without the FCGR2A H allele among patients with the KM 1 allele (i.e., difference between medians of left two plots) but not among patients without the KM 1 allele (difference between medians of right two plots).

Table 2 presents anti-PNAG IgG antibody levels for the study population, stratified by disease type. There were no significant differences in the distribution of antibody levels (*p* > 0.05) when compared across disease types using Kruskal–Wallis tests. Table 3 presents descriptive statistics (medians and interquartile ranges) for anti-PNAG IgG antibody levels, stratified by genotype groups. Significant differences between genotype groups are also noted, based on findings from QRMs examining the additive effect of the minor allele on IgG levels while adjusting for disease group. Results of the models suggest that most IgG antibody levels are not markedly different across the three genotypes for GM 3/17, GM 23, and KM. However, the levels of IgG4 antibodies to PNAG were associated with the additive effect of the minor allele (R) for *FCGR2A* genotypes (*p* = 0.01); the antibody levels were the highest among subjects homozygous for the R allele at this locus, lowest in those homozygous for the H allele, and intermediate in H/R heterozygotes. These results are depicted in Fig. 1. In addition to models that examined the main effects of the various genes, the QRMs used to investigate interactions between genes also yielded some interesting findings. Specifically, we identified a significant (*p* = 0.01) interaction between *FCGR2A* with KM 1/3 for IgG3; subjects with KM 1/1 or KM 1/3 and homozygous for the R allele of FCGR2A had the highest levels of anti-PNAG IgG3 antibodies compared to all other genotype combinations. These results are depicted in Fig. 2. In contrast to earlier report [8], KM allotypes were not associated with anti-PNAG IgG antibody responses in CF patients in this investigation (data not shown).

**Table 2:** Anti-PNAG IgG antibody levels for the study population, stratified by disease type^a^.

Variable	Statistic	Subjects with CF (*n* = 46)	Subjects with non-CF bronchiectasis (*n* = 150)	Subjects with other lung disease (*n* = 61)
IgG1 (AU/μL)	Median (IQR)	57.7 (41.5–87.1)	57.3 (33.4–99.4)	59.9 (42.1–94.0)
IgG2 (AU/μL)	Median (IQR)	161.9 (98.5–237.5)	185.0 (75.2–343.4)	217.9 (96.6–348.7)
IgG3 (AU/μL)	Median (IQR)	63.7 (39.2–97.9)	51.7 (23.0–101.6)	59.9 (39.3–95.1)
IgG4 (AU/μL)	Median (IQR)	40.4 (27.8–59.4)	32.5 (13.8–61.2)	35.4 (15.2–55.3)
Total IgG (AU/μL)	Median (IQR)	229.2 (148.0–339.2)	260.8 (153.4–334.6)	253.6 (182.9–313.1)

^a^There were no significant differences in the immunoglobulin G antibody levels (*p* > 0.05) when compared across disease types using Kruskal–Wallis tests.

*AU* absorbance unit, *IQR* interquartile range.

**Table 3:** Median anti-PNAG IgG antibody levels (and interquartile ranges), measured in absorbance unit/μL, stratified by genotype groups.

Gene	N	IgG1	IgG2	IgG3	IgG4	Total IgG
GM 3/17 genotype						
3/3	122	55.0 (34.0–81.1)	178.9 (91.5–294.8)	51.7 (24.6–84.8)	34.6 (15.6–61.2)	259.3 (181.6–326.2)
3/17	109	67.2 (34.6–99.8)	196.9 (84.6–342.7)	61.0 (32.4–104.1)	35.8 (18.6–55.4)	227.0 (148.2–328.2)
17/17	28	60.9 (43.0–133.9)	188.1 (74.0–311.9)	57.8 (32.2–80.6)	36.0 (18.8–62.6)	263.1 (138.5–330.9)
GM 23 genotype						
+/+	54	52.1 (32.6–8.00)	157.2 (89.4–276.2)	51.3 (28.2–80.4)	32.8 (17.0–56.0)	240.7 (139.6–317.6)
+/−	117	57.6 (37.4–87.1)	199.8 (101.6–334.2)	64.4 (31.8–99.8)	38.0 (18.8–60.2)	245.8 (170.4–328.2)
−/−	87	61.6 (36.8–107.8)	183.4 (73.0–305.1)	50.2 (26–93.5)	35.0 (15.6–59.4)	257.6 (145.6–331.6)
FCGR2A genotype						
H/H	63	55.6 (32.0–92.6)	199.2 (65.0–291.0)	46.7 (24.8–84.8)	24.8 (10.6–51.6)^a^	232.4 (122.2–305.0)
H/R	125	60.6 (38.6–95.3)	179.7 (89.4–335.3)	59.8 (29.4–97.9)	35.8 (18.8–56.0)^a^	246.6 (175.5–333.4)
R/R	71	57.0 (30.4–96.6)	179.0 (102.6–294.8)	57.0 (29.6–94.7)	41.6 (23.0–71.0)^a^	266.0 (153.9–330.2)
KM genotype						
1/1	6	65.8 (26.7–187.6)	242.0 (46.8–480.6)	62.8 (22.2–113.4)	28.1 (24.0–104.4)	325.2 (272.3–404.8)
1/3	36	55.5 (33.4–86.9)	139.2 (92.7–273.8)	52.4 (27.4–98.7)	29.2 (12.7–45.0)	224.1 (150.7–308.0)
3/3	217	57.6 (36.8–92.6)	191.2 (84.6–305.1)	55.0 (29.6–94.7)	37.0 (18.4–61.2)	247.6 (158.2–330.0)

^a^*P* < 0.05 based on quantile regression when comparing the median IgG antibody levels among the 3 genotype groups, examining the additive effect of the minor allele.

## Discussion

The most interesting finding in the current investigation is that *FCGR2A* alleles have an additive effect on the level of IgG4 antibodies to PNAG and an interactive effect with KM alleles on IgG3 antibodies to this polysaccharide. The mechanisms underlying these associations are not clear. IgG2, not IgG4, is usually a major IgG subclass in response to polysaccharide antigens. Because of its reduced binding affinity to activating FcγRs, IgG4 is a poor inducer of Fc-mediated effector functions, such as antibody-dependent cellular phagocytosis (ADCP) [13]. Therefore, association of the R allele of *FCGR2A* with high levels of IgG4 antibodies to PNAG observed in this investigation is unlikely to have a protective effect against the bacterial species which express PNAG. Interestingly, the R allele of *FCGR2A* has been reported to be risk a factor for acquiring chronic *Pseudomonas aeruginosa* infection in CF patients [9]. But the relevance of this report to the findings in this investigation is not clear, as PNAG expression has not been detected in *P. aeruginosa* [2]. IgG4, although the least abundant subclass, has several unique properties, not shared by other IgG subclasses [13]. For instance, IgG4 antibodies can exchange their arms in vivo, generating a hybrid antibody directed against two different antigens. This arms exchange phenomenon is influenced by IgG4 isoallotypes: an arginine at position 409 of IgG4 molecules enables the exchange, while a lysine at this position blocks it [14, 15]. To gain a deeper understanding of the relationship between *FCGR2A* polymorphism and IgG4 antibody responses to PNAG, it would be of interest to isolate affinity purified anti-PNAG IgG4 antibodies from the sera of PD patients, characterize their isoallotypes, and measure their affinity to allelically disparate FCGR2A molecules.

Subjects homozygous for the R allele of *FCGR2A* who were also KM 1-carriers had the highest levels of anti-PNAG IgG3 antibodies. Although IgG3 comprises the second lowest fraction of total IgG, it mediates very potent effector functions, such as ADCP, and it can also complex with C1q, activate classical complement pathway, and cause complement dependent cytotoxicity (CDC) of infectious pathogens [16]. Both R- and H-expressing FCGR2A molecules appear to have similar affinities for IgG3 antibodies [10] and could contribute to the IgG3-meditaed ADCP and CDC of PNAG-expressing bacteria. The lack of association between KM allotypes and anti-PNAG antibody responses in CF patients here—in contrast to the earlier report [8]—may be due to a smaller sample size of CF patients in this investigation (46 vs 58). Also, CF patients in the earlier investigation were children, whereas the patients in the present investigation were adults.

KM 1 allotype has been associated with high antibody responsiveness to *Haemophilus influenzae* type b vaccine [17]. The anti-capsular antibody responses were significantly higher in KM 1-carriers than in subjects who lacked this determinant. Heterozygosity at the KM locus has been associated with humoral immunity to *Campylobacter jejuni* [18]. Both *Haemophilus influenzae* and *Campylobacter jejuni* express PNAG [2]. How KM and *FCGR2A* alleles epistatically interact and contribute to anti-PNAG IgG3 antibody responses is not clear. KM alleles have been shown to interact with other genes of the immune system and contribute to immunity to particular antigens. For instance, they interact with GM alleles and contribute to the cellular immune responses to streptococcal cell wall antigen [19] and interact with HLA alleles in anticentromere antibody production [20].

This study has certain limitations. The study cohort was predominantly white. Since there are racial differences in gene frequencies, the findings reported here may not be applicable to other racial/ethnic groups. Also, we do not have complete data on some PD and treatment-related factors, which potentially could have influenced antibody responsiveness to PNAG.

There is a paucity of gene-gene interaction studies in immunobiology in general. Importance of PNAG as a potential vaccine candidate against numerous infectious pathogens warrants large multiethnic studies, involving many genes of the immune system, to help identify factors that influence immunity to this polysaccharide.

## Data Availability

The data generated in this study will be available from the corresponding author upon reasonable request.
